# EHR problem list clustering for improved topic-space navigation

**DOI:** 10.1186/s12911-019-0789-9

**Published:** 2019-04-04

**Authors:** Markus Kreuzthaler, Bastian Pfeifer, Jose Antonio Vera Ramos, Diether Kramer, Victor Grogger, Sylvia Bredenfeldt, Markus Pedevilla, Peter Krisper, Stefan Schulz

**Affiliations:** 1 Institute for Medical Informatics, Statistics and Documentation, Medical University of Graz, Graz, Austria; 2 KAGes Steiermärkische Krankenanstaltengesellschaft m.b.H, Graz, Austria; 3 Division of Nephrology and Dialysis, Department of Internal Medicine, Medical University of Graz, Graz, Austria; 4 CBmed GmbH – Center for Biomarker Research in Medicine, Graz, Austria

## Abstract

**Background:**

The amount of patient-related information within clinical information systems accumulates over time, especially in cases where patients suffer from chronic diseases with many hospitalizations and consultations. The diagnosis or problem list is an important feature of the electronic health record, which provides a dynamic account of a patient’s current illness and past history. In the case of an Austrian hospital network, problem list entries are limited to fifty characters and are potentially linked to ICD-10. The requirement of producing ICD codes at each hospital stay, together with the length limitation of list items leads to highly redundant problem lists, which conflicts with the physicians’ need of getting a good overview of a patient in short time.

This paper investigates a method, by which problem list items can be semantically grouped, in order to allow for fast navigation through patient-related topic spaces.

**Methods:**

We applied a minimal language-dependent preprocessing strategy and mapped problem list entries as *tf-idf* weighted character 3-grams into a numerical vector space. Based on this representation we used the unweighted pair group method with arithmetic mean (UPGMA) clustering algorithm with cosine distances and inferred an optimal boundary in order to form semantically consistent topic spaces, taking into consideration different levels of dimensionality reduction via latent semantic analysis (LSA).

**Results:**

With the proposed clustering approach, evaluated via an intra- and inter-patient scenario in combination with a natural language pipeline, we achieved an average compression rate of 80% of the initial list items forming consistent semantic topic spaces with an F-measure greater than 0.80 in both cases. The average number of identified topics in the intra-patient case (*μ*_*Intra*_ = 78.4) was slightly lower than in the inter-patient case (*μ*_*Inter*_ = 83.4). LSA-based feature space reduction had no significant positive performance impact in our investigations.

**Conclusions:**

The investigation presented here is centered on a data-driven solution to the known problem of information overload, which causes ineffective human-computer interactions at clinicians’ work places. This problem is addressed by navigable disease topic spaces where related items are grouped and the topics can be more easily accessed.

## Background

Through lifelong and nationwide Electronic Health Record (EHR) systems, larger and larger amounts of patient information will be available at clinicians’ workplaces. Flooding the user which highly granular and partly redundant information is especially relevant when patients have chronic diseases, multiple diagnoses and numerous in- and outpatient treatment episodes.

This circumstance hampers a quick overview of the most important facts, possibly with a negative influence on the quality of medical decisions. For a long time, problem lists or diagnosis lists in medical records have been key information sources, because they contain a palatable selection of the most relevant information items, filtered and summarized by physicians.

In the setting in which this study is embedded, i.e. in a large Austrian hospital network, the clinical information system displays problem list entries up to 50 characters only. Furthermore, problem lists are, first of all, diagnosis lists, and each coded diagnosis at each hospital stay produces a new problem list entry. Due to the length limitation of list items, most official ICD labels are overwritten by the users, often drastically abbreviated and enriched by additional information like time or other contexts of a diagnosis.

To improve the access of physicians to problem list entries, especially by reducing redundancy is the main objective of a so-called patient-centered QuickView mode we have developed and deployed via a web-based front-end from of the clinical information system i.s.h.med. Whereas the ultimate goal of QuickView is a navigable, user-centered overview of a patient’s diseases, medications, procedures and laboratory results, we here limit ourselves to a problem list like diagnosis lists, most of which coded by ICD-10. Such lists easily amount to a length of hundreds of items for elderly or multi-morbid patients. We intend to provide a topic-based grouping, which can be exploited in a navigational and information visualization based way within QuickView.

Analyzing EHR content with supervised and unsupervised machine learning methods has become a widely used approach to gain insights into clinical information like diagnoses [1] or medications [2–5], and at the same time it is also a matter of investigation in different academic challenges [6].

Information extraction from unstructured EHR data like clinical narratives is a general challenging task, due to language specific idiosyncrasies like short forms (abbreviations [7, 8], acronyms [9, 10]), spelling and typing mistakes, syntactic incompleteness, specialist jargon, negations [11] or non-standardized numeric expression, just to mention some [12, 13]. The automatic assignment of ICD diagnosis codes received special attention in various research projects due to its importance for therapy planning, billing and medical decision support.

Koopman et al. [14] used support vector machines (SVMs) with term and concept based features to automatically detect cancer diagnoses and classify them according to ICD–10. An F-measure of 0.70 was reported for detecting the type of cancer. Koopman et al. [15] also automatically classified death certificates with respect to influenza, diabetes, pneumonia and HIV. A supervised approach with SVMs was used for ICD-10 coding, resulting in an F-measure of 0.80. Ning et al. [16] tested a Chinese ICD-10 coding approach on medical narratives. Based on a word-to-word similarity metric, they structured the ICD-10 codes hierarchically and assigned codes to unlabeled documents with an F-measure of 0.91. Chen et al. [17] enhanced the longest common subsequence algorithm for ICD-10 mapping to Chinese clinical narratives, yielding an F-measure of 0.81 for this task. Boytcheva [18] achieved an F-measure of 0.84 using a multi-class SVM with a max-win voting strategy in combination with a text preprocessing module for ICD-10 coding of Bulgarian clinical narratives.

However, features used in a supervised framework are often connected to language-specific patterns, even though more recent deep learning methods reduce the need for use case specific feature engineering e.g. for clinical narrative de-identification [19, 20].

In the following sections we will present and evaluate a minimal language-dependent approach of semantic grouping of problem list entries, without the need of human feature engineering. We refrain from a purely supervised approach, but will use a post-ICD-10 coding methodology with the side effect that documents where no code could have been assigned are nevertheless grouped together in semantically meaningful clusters.

## Methods and materials

### Intra-patient data-set

For intra-patient inspection, we used data from five de-identified nephrology patients, each of them having between 250 and 861 50-character long problem list statements written in German, covering time intervals from 12 to 22 years. A special feature of these code-description pairs is the fact that physicians can overwrite the contents of a 50-character long text field originally filled with standardized text generated by an ICD-10 coding plug-in. The list view therefore consists of different standardized and personalized diagnosis entries, the latter often being enriched with additional context like time references, procedures, or medications. Additionally, ICD-10 codes with no textual description as well as entries without ICD-10 codes occur. This makes these lists, originally devised as ICD-based *diagnosis lists*, resemble *problem lists*, a feature rooted in Anglo-Saxon medical traditions, but uncommon in German-speaking clinical communities.

### Inter-patient data set

We used the sampling theorem with Chernoff bounds [21, 22] in order to estimate a statistical representative sample size for nephrology patients for the *inter-patient* inspection:

1 $$ n\ge \frac{3}{\varepsilon^2}\kern0.5em In\kern0.5em \frac{2}{\delta } $$

With an accuracy of *ε* = 0.05 and a confidence of 1 − *δ* = 0.95, 4430 non-identical ICD-10 coded de-identified 50-character long text snippets were chosen as a representative linguistic sample size (4430 ≥ *n* = 4427). The advantage of using the sampling theorem is its independence of the overall initial pool size for estimating a number of samples. By applying this theorem, we claim that a representative syntactical pattern of the sampled corpus, in our case the non-identical short ICD-10 code descriptions, with a probability of 95%, is within +\- 5% of the overall observations. With this approach for sub sample size estimation we addressed a significant amount of linguistic variations in a clinical domain, for *inter-patient* post-ICD-10 encoding. Finally, we merged the five de-identified patients from the *intra-patient* pool with the 4430 ICD-10 samples.

### Problem description

A patient *P*_1..*i*_ has a set of diagnosis list items *I*_1..*k*..*l*_ where *I*_*k*_ = (*ICD* − 10_*k*_, **d**_*k*_) defines the 50-character long description *d*_*k*_ which we refer to as a *document* in the following analysis. One fraction *I*_*coded*_ = *I*_1..*k*_ is coded and the other one *I*_*uncoded*_ = *I*_*k* + 1..*l*_ is without codes, with just the text snippets **d**_*k* + 1..*l*_ existing. Since an immediate overview of all list items *I*_1..*l*_ to a patient *P*_*i*_ is not possible with longer lists, our solution attempts to semantically group them into *n* sets *C*_1..*n*_, so that the content navigation through all list items *I*_1..*l*_ via *C*_1..*n*_ is supported.

For semantically grouping related list items *I*_1..*l*_, we make use of the fact that list items *I*_*coded*_ with the same 3-digit ICD-10 code are similar in content. Existing codes to a document form a manual ground truth of judgment for semantic similarity. On the other hand, content similarity of a subgroup of list items *I*_*i*..*j*_ out of *I*_1..*l*_ is given by string similarity between two list items (*I*_1_, *I*_2_), which can be expressed via a function *f*_*sim*_(*I*_1_, *I*_2_) = *sim* = *f*_*sim*_(**d**_1_, **d**_2_). Therefore *sim* is an indicator for content similarity.

In cases where list items have the same ICD-10 code, we clustered them forming *C*_*ICD* − 10_ = *C*_1..*i*_ ICD-10 content groups. Therefore we tried to post-assign ICD-10 codes to the uncoded list items *I*_*uncoded*_ while those list items which got no post-ICD-10 code assigned could at least be grouped as being similar in content, via a certain level of *sim* forming *C*_*sim*_ = *C*_*i* + 1..*n*_ cluster. We therefore evaluated the correct post-ICD-10 assignment of list items in *C*_*ICD* − 10_ and the correct clustering of content groups *C*_*sim*_ where no code could be assigned based on string similarity.

We aimed to achieve this *in one go* by using a hierarchical clustering approach wherever ICD-10 codes are assigned to non-coded list items and at the same time infer the optimal *sim* boundary for string-based list item grouping with a minimal language-dependent preprocessing strategy. We apply the methodology in an *intra-patient* and an *inter-patient* scenario. For *inter-patient* post-ICD-10 assignment we assumed that the number of assigned ICD-10 codes was significantly higher compared to the *intra-patient* scenario, due to the fact that codes can be assigned via learning from examples of other patients.

### Evaluation methodology

We use the metrics *Precision* = #TPs / (#TPs + #FPs), *Recall* = #TPs / (#TPs + #FNs) and *F-measure* = 2 · *Precision* · *Recall* / (*Precision* + *Recall*) [23], in order to evaluate the accuracy of our topic groups *C*_1..*n*_, for the *intra-patient* and for the *inter-patient* approach, respectively. True Positive (TP): A topic gets *correctly* assigned. False Positive (FP): A topic gets *incorrectly* assigned. False Negative (FN): A topic *should have been* assigned. True Negative (TN): A topic was *correctly not* assigned. A topic can be specified via a specific *3-digit* ICD-10 code or a certain content cluster in case it is not possible to assign a post-ICD-code description.

### Data preprocessing

**Fig. 1:**
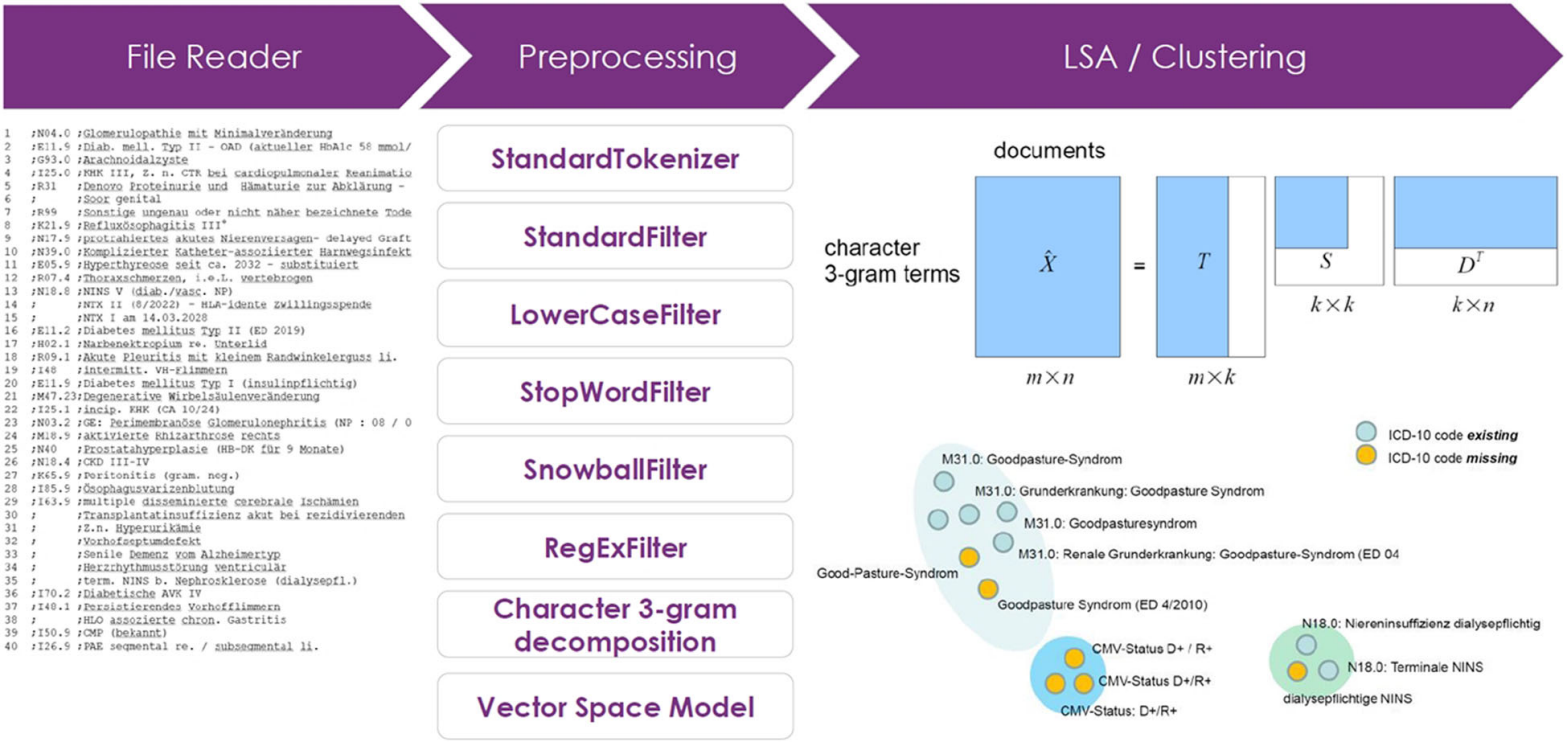
Overview of the main data flow and preprocessing steps until the selected clustering approach has been applied at the end of the overall processing chain

The 50-character text segments were normalized using the following Lucene [24]-based NLP processing chain: a *StandardTokenizer* for tokenizing the very short narratives; a *StandardFilter* applying a base orthographic normalization; a *LowerCaseFilter* to eliminate all upper case occurrences; a *StopWordFilter* erasing a list of defined tokens and a *SnowballFilter* (“German2”) for stemming (Fig. 1). Finally a specific set of characters were removed from the normalized token stream via a specific regular expression ([\d\.\,\_\:]+). We compensated the especially German language specific phenomenon of word compounds, e.g. certain domain-specific affixes like “-itis” for inflammation or “-ektomie” for surgical removal, not by a specific word decompounding engine but by a character n-gram filter, choosing an initial window size of *n* = 3. The side effect of character *n-gram* modeling is that typing errors, commonly found in clinical narratives have less impact on token dissimilarity in the VSM (Vector Space Model).

### Vector space model

We mapped the EHR problem list items into a vector space using the VSM [25, 26] which models a set of documents *D* = **d**_1_, **d**_2_, **d**_*j*_, … , **d**_*n*_ as bag of words where a document **d**_*j*_ defines a point in the *m*-dimensional vector space, forming an *m*-dimensional feature vector. The dimensionality *m* of the feature space in our case is defined via *t*_1_, *t*_2_, *t*_*i*_, … , *t*_*m*_ unique character 3-gram types of the preprocessed document collection *D* and the VSM is therefore described via a *m*×*n* matrix **X**. We applied the term frequency – inverse document frequency *tf-idf* weighting scheme on **X** and used the cosine similarity between two documents *d*_*i*_ and *d*_*j*_ to obtain the semantic similarity *sim* between two list items *I*_*i*_ and *I*_*j*_.

### Latent semantic analysis

We examined Latent Semantic Analysis (LSA) and different degrees of dimension reduction of the semantic space for its impact on our topic model approach. The mathematical core function of LSA [27, 28] is a Singular Value Decomposition (SVD) of the term-document matrix **X = TSD**^*T*^ accessing the orthonormal matrices **T** and **D**^*T*^ with the eigenvectors of **XX**^*T*^ and **X**^*T*^**X. T** defines the term matrix and **D**^*T*^ the document matrix. The roots of the eigenvalues of **XX**^*T*^ and **X**^*T*^**X** are embedded in **S**. The degree of dimensionality reduction can be controlled by eliminating the lowest eigenvalues and their eigenvectors to a new dimension *k* resulting in a dimensionality reduced space **T**_*k*_ respectively **D**^***T***^_*k*_. The orthonormal semantic spaces **T**_*k*_, **D**^***T***^_*k*_ can be seen as one kind of distributional semantics and are exploited in various information retrieval and information extraction scenarios.

### Clustering methodology

For content-based grouping into *n* sets *C*_1..*n*_ we applied a clustering approach. First, for all patient-specific documents *d*_1..*l*_ (50-character long phrases) including the already ICD-10 coded documents we applied a hierarchical agglomerative cluster method implemented in the R package *fastcluster* [29]. In brief, agglomerative clustering works as follows: All documents are initially assigned to their own cluster and then iteratively merged, based on a specific distance metric until there is just a single cluster. To decide whether two cluster collapse into a single one we used the *Unweighted Pair Group Method with Arithmetic Mean* (UPGMA) variant. It computes the distances between two cluster *C*_*1*_ and *C*_*2*_ based on the pairwise average distances between their assigned documents *d*:

2 $$ \frac{1}{\left|{C}_1\right|\left|{C}_2\right|}\kern0.5em {\sum}_{{\mathbf{d}}_i\in {c}_1}{\sum}_{{\mathbf{d}}_j\in {c}_2}\left(1-{f}_{sim}\left({\mathbf{d}}_i,{\mathbf{d}}_j\right)\right) $$

We hypothesize that string similarity of textual problem list entries (i.e. the documents) correlate with their ICD-10 code assignments, therefore we expect that UPGMA in combination with the chosen cosine similarity distance metric delivers good results. We applied different cut heights to the resulting dendrogram and inferred the cut-off (cut-height of the dendrogram) that most accurately reproduced the already coded ICD-10 clustering scheme (*I*_*coded*_). A big advantage of the UPGMA clustering is that we can directly relate the resulting clusters to the cosine distances between the documents whereas other algorithms like k-means for example require a pre-defined parameter *k* for the number of clusters. Accuracy was estimated by the F-measure for the *intra-* as well as the *inter-patient* scenario.

In fact, one could also infer an appropriate cut-off based on more conservative approaches like the *Elbow* [30] or *Silhouette* [31] method to enable a purely unsupervised setting. However, in our framework these methods would separate clusters exclusively based on string similarity, which may not capture the true n-gram variances within the semantic clusters and as consequence will likely produce a high *false negative rate*.

3 $$ ICD-{10}_c\left({I}_{uncoded}\right)= ICD-{10}_c\left(\max \left\{{f}_{sim}\left({\mathbf{d}}_l,{\mathbf{d}}_k\right)\right\},{I}_{coded}\right) $$

Equation 3 gives a formal explanation of how the post coding of ICD-10 codes was executed. Unlabeled documents (**d**_*l*_ ∈ *I*_*uncoded*_) were coded if and only if they appeared in a same cluster *C* (**d**_*l*_, **d**_*k*_ ∈ *C*) together with at least one ICD-10 coded document (**d**_*k*_ ∈ *I*_*coded*_). In cases where documents with different ICD-10 codes were clustered in the same group, we assigned the label of the document with the smallest cosine distance transforming the diagnosis into a coded list item.

## Results and discussion

We used a hierarchical clustering approach to semantically cluster EHR problem lists, where semantic similarity was specified by ICD-10 codes and string similarity. The main challenge of this approach is to find the optimal cut-off height of the resulting dendrogram to ensure optimal post-ICD-10 coding and reasonable string clustering at the same time. With the hypothesis that ICD-10 coding correlates with string similarity we were able to exploit the already coded 50-character as a reference for this optimization problem.

Specifically, we inferred a cut-off such as the coded 50-character long diagnosis texts with the same 3-digit ICD-10 code fall into the same grouping based on string similarity. This is achieved by iteratively applying different cut-off heights and finally choose the one with the maximum F-measure. For this study we report an averaged intra-patient F-measure of 0.70 at a cut-off height 0.90 for patients *P*_1..5_ and an F-measure of 0.47 at a cut-off height 0.97 for the inter-patient approach. From these first results we could conclude that our assumption exclusively holds for a subset of diagnosis lists reflecting an ICD-10 cluster (intra-patient). Re-sampling a fully representative character 3-gram distribution (inter-patient) of the ICD-10 specific diagnosis texts strongly discard this assumption due to the high variances observed within the ICD-10 groups. However, while the obtained cut-off purely performs in detecting *true negatives* it does remarkable well in post-assigning ICD-10-codes.

**Fig. 2:**
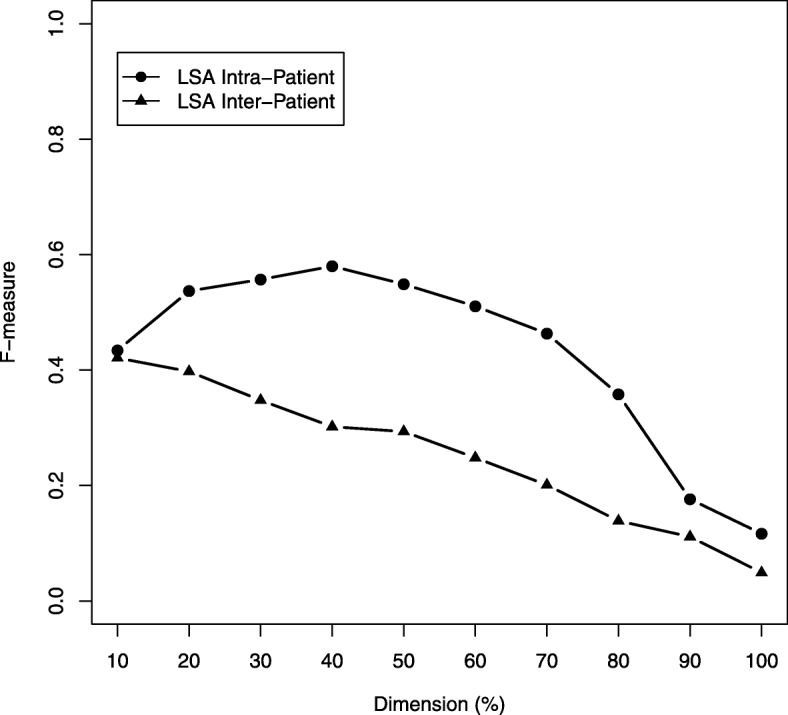
Averaged step-wise *intra*-patient and *inter*-patient dimension reduction of the semantic document space

In an additional investigation, as depicted in Fig. 2, we inspected the influence of transforming the character 3-gram term-document matrix **X** into its semantic orthogonal document space **D**^***T***^_*k*_ and varied the dimension reduction at *k* different levels. We observed a maximum F-measure of 0.58 using 40% of the most relevant dimensions for the *intra-patient* case and an F-measure of F = 0.42 with the 10% of the most relevant dimensions for the *inter-patient* case. Thus, mapping the problem into a reduced linear transformed semantic space via LSA not yet improved the performance of our approach.

Table 1 highlights the results for the *intra-patient* post-ICD-10 coding at the top and the string clustering results at the bottom. On average 68% of the non-coded list items were post assigned with an F-measure of 0.77. The remaining 32%,where no ICD-10 code could have been assigned, formed consistent topic clusters with an F-measure of 0.85. We therefore report an overall list item grouping for the *intra-patient* inspection with an F-measure of 0.81.

**Table 1:** *Intra-patient* post-ICD-10 coding and string clustering results

Patient	Coded	Precision	Recall	F-measure
P_1_	0.67	0.93	0.74	0.83
P_2_	0.60	0.90	0.61	0.73
P_3_	0.68	0.73	0.69	0.71
P_4_	0.87	0.91	0.87	0.89
P_5_	0.59	0.80	0.63	0.70
Patient	Clustered	Precision	Recall	F-measure
P_1_	0.33	0.78	1.00	0.88
P_2_	0.40	0.91	0.78	0.84
P_3_	0.32	0.84	0.81	0.82
P_4_	0.13	1.00	1.00	1.00
P_5_	0.41	0.59	0.93	0.72

From Table 2 we see that for the *inter-patient* setting almost all non-coded list items get a post-assigned ICD-10 code with an overall F-measure of 0.87. This result is quite remarkable compared to the literature review and considering the not optimal cut-off we inferred for the *inter-patient* inspection accomplished by a lower precision compared to the *intra-patient* results in Table 1. However, the expected recall gain had an overall positive performance impact judged by the F-measure.

**Table 2:** *Inter-patient* post-ICD-10 coding

Patient	Coded	Precision	Recall	F-measure
P_1_	1.00	0.76	1.00	0.86
P_2_	0.99	0.85	0.99	0.91
P_3_	0.99	0.75	1.00	0.86
P_4_	1.00	0.78	1.00	0.88
P_5_	0.99	0.70	1.00	0.82

The post-ICD-10 coding rate is indeed that high that the portion of list items without code has no relevant impact on the overall topic groups *C*_1..*n*_, to support the navigation through all list items *I*_1..*l*_ via *C*_1..*n*_. We therefore report an overall list item grouping for the *inter-patient* inspection with an F-measure of 0.87 mainly dominated by ICD-10 codes.

Tables 3 and 4 show that the number of identified topics on average in the *intra-patient* case (*μ*_*Intra*_ = 78.4) was lower than in the *inter-patient* case (*μ*_*Inter*_ = 83.4) as well as initial list items views like in the case of Patient 3 with more than 850 entries can be semantically grouped to less than 100 entry points. This is equivalent to a semantic compression rate of up to 89% of the original list item size.

**Table 3:** Number of the identified *intra-patient* topics out of the initial disease list items

Patient	List items	Unique list items	Topics	Compression rate
P_1_	302	184	60	0.80
P_2_	250	174	70	0.72
P_3_	861	441	95	0.89
P_4_	531	295	77	0.85
P_5_	378	262	90	0.76

**Table 4:** Number of the identified *inter-patient* topics out of the initial disease list items

Patient	List items	Unique list items	Topics	Compression rate
P_1_	302	184	61	0.80
P_2_	250	174	65	0.74
P_3_	861	441	118	0.86
P_4_	531	295	82	0.85
P_5_	378	262	91	0.76

Despite the good results of our approach two major challenges need to be addressed: i) Some textual expressions should be coded with more than one ICD-10 code. For instance, in the case of “Akutes Nierenversagen mit Hyperkaliämie” (acute kidney failure with hyperkalaemia) N17 (acute renal failure) should be assigned to “Akutes Nierenversagen” (acute kidney failure) and E87 (other disorders of fluid, electrolyte and acid-base balance) for “Hyperkaliämie” (hyperkalaemia). So far we have inferred exactly one code per 50-character list entry. ii) Some codes were found to be plainly wrong at the moment we post-assign the codes at the quality level of clinical routine documentation.

## Conclusions

In this paper we have motivated a hierarchical cluster-based approach with a minimal language-dependent preprocessing strategy for grouping clinical problem lists into distinct semantically similar clusters in order to support patient-based disease topic navigation. This functionality is planned to be implemented within a QuickView software accessible in a hospital environment.

Our methodology not only post-assigns ICD-10 codes but also builds semantically similar clusters based on string similarity. Applying this method at an *intra-patient* level implies that possible post-ICD mappings are missing due to the limited patient-focused scope (high *false negative rate*), nevertheless achieving a useful clustering of list-items where no code could be assigned. For this reason, we extended the scope to an *inter-patient* examination of the same methodology and motivated a sufficient sample size in order to fetch a common linguistic fingerprint. With an acceptable negative impact on precision we were able to boost recall so that the overall topic modeling of the disease space was reduced to post-ICD-10 codes only. However, the *inter-patient* cut-off height of the resulting dendrogram is at a very low level, with the result that the *inter*-cluster variance is not at its optimal state anymore with regard to string similarity. As a consequence, a substantial amount of list items gets ICD-10 code assigned by accident.

In a further investigation we plan to refrain from an F-measure driven optimized *single* cut-off strategy, and want to pursue a strategy where the ICD-10 cluster-specific variances on our proposed normalized character 3-gram features can be studied more reliably. In this case also a more detailed inspection of the level of character n-gram decomposition could be done. We hypothesize that, while estimating the optimal number of disease clusters based on a between-within variance inspection, already encoded ICD-10 examples can just act as proxies for correct post-ICD encoding and therefore may compensate for the precision loss at a high recall level. One avenue would be a more conservative method like *Elbow* and *Silhouette* to infer the best cut-off purely based on string similarity and dynamically encode potentially *false negatives* in a post-processing step where each ICD-10 cluster is treated independently based on their feature pattern space respectively character *n-gram* distribution.

## Abbreviations

EHR: Electronic Health Record
HIS: Hospital Information System
ICD-10: 10th revision of the International Statistical Classification of Diseases and Related Health Problems
LSA: Latent Semantic Analysis
SVM: Support Vector Machine
VSM: Vector Space Model
